# Pregnancy loss and Income in the Republic of Korea using National Health Insurance Service Data, 2008–2014

**DOI:** 10.1186/s12889-022-12588-0

**Published:** 2022-01-27

**Authors:** Ga-Young Lim, Na Young Jung, Kyo Yeon Jun, Ji Yeon Kang, Mi Kyung Kim, Hye-Eun Lee, Myoung-Hee Kim, Jaechul Song, Inah Kim, Yu-Mi Kim

**Affiliations:** 1 Department of Public Health, Hanyang University Graduate School, Seoul, South Korea; 2 Center for Cohort Studies, Total Healthcare Center, Kangbuk Samsung Hospital, Sungkyunkwan University School of Medicine, Seoul, South Korea; 3 Incheon Communicable Diseases Center, Incheon, South Korea; 4 Occupational Safety and Health Research Institute, Korea Occupational Safety and Health Agency, Incheon, South Korea; 5 Korea National Enterprise for Clinical Trials, Seoul, South Korea; 6 Department of Preventive Medicine, Hanyang University College of Medicine, 222 Wangsimni-ro, Seongdong-gu, Seoul, 04763 South Korea; 7 Hanyang University School of Public Health, Seoul, South Korea; 8 Korea Institute of Labor Safety and Health, Seoul, South Korea; 9 Department of Social and Preventive Medicine, Hallym University College of Medicine, Chuncheon, South Korea; 10 Research Institute of Public Health, National Medical Center, Seoul, South Korea; 11 Department of Occupational and Environmental Medicine, Hanyang University College of Medicine, Seoul, South Korea

**Keywords:** Pregnancy outcome, Miscarriage, Stillbirth, Prevalence, Income, Republic of Korea

## Abstract

**Background:**

Although unintentional pregnancy loss is common, national representative statistics are lacking in high-income East Asian countries undergoing rapid demographic changes. It is necessary to confirm the income inequality of pregnancy loss even in universal national health insurance.

**Method:**

Using National Health Insurance Service data between 2008 and 2014, the annual prevalence of pregnancy loss was enumerated, and differences in pregnancy loss according to age and income levels were assessed by multivariable Poisson regression. Joint-point regression was used to examine the trend of pregnancy loss.

**Result:**

On average, there was a 15.0% annual pregnancy loss among 3,941,020 pregnancy cases from 2008 to 2014. Pregnancy loss inequality increased stepwise with income levels except for the highest income group. After adjusting for income levels, the annual percent change of age-standardized prevalence significantly increased by 2.6% every year since 2011.

**Conclusion:**

Even in high-income countries with universal national health insurance, income inequality in pregnancy loss is observed. Further appraisal is needed to explain the increasing trend of pregnancy loss between 2011 and 2014 even after adjusting income.

## Introduction

Pregnancy outcomes are important issues in women’s reproductive health, including livebirths, stillbirths, spontaneous abortions, and induced abortions. About 22% of embryo implantations was known to end before clinically detection of pregnancy [1]. Approximately 80% of pregnancy loss was during the first 12 gestational weeks [2]. The criterion for distinguishing a stillbirth from a miscarriage, also referred to as spontaneous abortion, is fetal viability, which is generally based on 20 to 24 gestational weeks. In high-income countries, stillbirth is supposedly caused by placental dysfunction and very early preterm birth, with approximately 0.5% of pregnancies reaching 22 gestational weeks [3].

Pregnancy loss rates varies in the Republic of Korea, which is inconsistent with the results of previous studies. According to the Korea National Fertility and Family Health and Welfare Survey, one-fifth of pregnant women experienced pregnancy loss. However, this survey was only targeted at married women [4]. In the 1990s, a follow-up study based on a community found 586 pregnancies and confirmed 452 pregnancy outcomes, with 33 fetal deaths (7.0%) [5]. Using the national health survey data from 2010 to 2012, the frequency of self-reported spontaneous abortion of 3260 married women who experienced pregnancy more than once was 0.344 ± 0.705 times [6].

The age of parents has been identified as a significant influencing factor, and the risk of spontaneous abortion increased rapidly, especially at the maternal age of 35 years [7, 8]. The ages of both the parent are related to spontaneous abortion [9–11]. In the Republic of Korea, with the recent sharp reduction in the reproductive age, marriages and childbirths have decreased in all age groups for seven consecutive years since 2012. The mean age of primiparous mothers increased from 29.6 years in 2008 to 31.8 years in 2018 [12]. However, social reproduction and women’s reproductive health indicators at the national level only focus on live births, including the number of births and fertility rates.

Not only pathophysiologic factors such as chromosomal abnormalities [13, 14] and maternal illness history including recurrent miscarriage [15, 16], environmental factors such as long working hours or shift work [17, 18] and handling heavy objects [19], and chemicals [20–22] could also increase the risk of miscarriage. Although low socioeconomic position is proposed as a risk factor for pregnancy loss [16, 23–26], it is necessary to confirm whether there is a socioeconomic inequality in high-income East Asian countries with universal national health insurance coverage.

Therefore, we aimed to describe the current status of pregnancy loss and assessed the pregnancy loss gap across income levels using the National Health Insurance Service-National Sample Cohort (NHIS-NSC), which is a population-based cohort established by National Health Insurance Service (NHIS) in the Republic of Korea. Additionally, the trend of pregnancy loss from 2008 to 2014 was examined.

## Methods

### Data and study population

The NHIS-NSC, which is the Korean nationwide health care services dataset from 2008 to 2014, was used in this study. Over 97% of Koreans are enrolled in NHIS institutionalized for mandatory subscription [27].

The Korean Standard Classification of Diseases (KCD-6) code adapted by the NHIS was used to identify pregnant cases and pregnancy loss. Operationally, a pregnancy case was a person whose pregnancy outcome was confirmed by the pregnancy termination treatment code during pregnancy diagnosis. From January 2008 to December 2014, 5,451,613 cases were identified using the pregnancy diagnosis code: the overall O codes (pregnancy, childbirth, and postpartum care), Z32.1 (confirmed pregnancy), Z34 (prenatal care of normal pregnancy), Z35 (prenatal care of high-risk pregnancy), and Z36 (prenatal screening). A total of 193,291 cases with missing age variables, under the age of 20 years, or over the age of 50 years were excluded.

The pregnancy outcome types were confirmed based on “medical treatment DB”, and the pregnancy-related disease codes were identified through medical statements, medical care usage, and medical histories. Only participants with delivery (O80 – O84), spontaneous abortion (O03, O02.1) and stillbirth (Z37.1, Z37.3, Z37.4, Z37.6, Z37.7) were included. This resulted in the exclusion of 164,756 cases of artificial abortions (O04, O05, O06) and ectopic pregnancies (O00). A total of 1,087,183 participants with confirmed pregnancies but without confirmed termination statuses were also excluded. After excluding 65,363 cases with errors in insurance rating information, 3,941,020 pregnancy cased were finally included.

This study conducted in accordance with World Medical Association Declaration of Helsinki. The Hanyang University Institutional Review Board approved this study (IRB number: HYI-17-215-2).

### Age and income variables

As age and socioeconomic position has been considered important factors in pregnancy loss, we sought to obtain those factors from the NHIS-NSC.

Age and income variables were identified using the National Health Information Database (NHID) healthcare use database and the eligibility database. Age variables were categorized by 5 years from 20 to 49 years old. National health insurance premium grades obtained from the NHID eligibility data imposed proportionally based on monthly salary as a measure of income. These premium grade indicators, which were ordered up to 20-quantiles, have been used in epidemiologic studies of health inequalities across income levels [28, 29]. Income levels are classified as Q0 (Medical Beneficiary), Q1 (the lower class, grades 1st – 5th), Q2 (the lower-middle class, grades 6th – 10th), Q3 (the upper-middle class, grades 11th – 15th) and Q4 (The upper class, grades 16th – 20th).

### Pregnancy loss

Pregnancy outcomes were classified as delivery (O80 – O84), stillbirth (Z37.1, Z37.3, Z37.4, Z37.6, Z37.7), and spontaneous abortion (O03, O02.1) according to the KCD-6. The final outcome variable was “pregnancy loss (stillbirth or spontaneous abortion code)”.

### Statistical Analysis

Cross tabulation and Mantel–Haenszel chi-square test were used to examine the distribution consistency of operationally defined pregnancy and pregnancy loss according to age and income levels yearly.

We calculated the annual prevalence of pregnancy loss as the number of pregnancy losses with a specified pregnancy termination code in a year, divided by the number of operationally defined pregnancy cases that year. The average annual prevalence for 7 years was calculated using the overall number of pregnancy losses from 2008 to 2014 as the numerator and the number of operationally defined pregnant cases during this period as the denominator. Crude and adjusted prevalence ratios according to age and income levels were estimated using multivariable Poisson regression analysis with the PROC GENMOD procedure [30]. The prevalence ratio according to age and income levels were estimated based on the following equations:

Log (number of pregnancy losses) = Log(number of operationally defined pregnant cases) + β_0_ + β_11_ * Age group I (20-24 yrs) + β_12_ * Age group III (30-34 yrs) + β_13_ * Age group IV (35-39 yrs) + β_14_ * Age group V (40-44 yrs) + β_15_ * Age group VI (45-49 yrs) + β_21_ * Income group I (Q0, lowest) + β_22_ * Income group II (Q1) + β_23_ * Income group III (Q2) + β_24_ * Income group V (Q4, highest).

Joint-point regression analysis was used to identify trends in annual percent change (APC) of age-standardized prevalence of pregnancy loss from 2008 to 2014. Joint-point Regression Program version 4.1.0 (US National Cancer Institute, Bethesda, MD, USA) was employed for this. The number of people for each age group (from 2008 to 2014) was set as the standard population for direct age standardization.

## Results

The annual prevalence of pregnancy loss and distributions of operationally defined pregnancy cases according to age and income levels of the 3,941,020 study subjects are presented in Table 1. From 2008 to 2014, a total of 591,127 pregnancy losses accounted for 15.0% of the operationally defined pregnancy cases. The annual pregnancy loss, which was stagnant from 2008 to 2011, has increased since 2012. The difference in yearly distribution was significant (P for trend by Mantel–Haenszel test < 0.0001). The proportion of women aged over 35 years was 12.1% in 2008 and 17.7% in 2014 (P by Mantel-Haenszel test < 0.0001).

**Table 1:** Annual crude prevalence of pregnancy loss and distributions of operationally defined pregnant cases according to age and income levels between 2008 and 2014

	2008		2009		2010		2011		2012		2013		2014		*P*-value
	N	(%)	N	(%)	N	(%)	N	(%)	N	(%)	N	(%)	N	(%)	
															<.0001^a^
Pregnant case	575,382		568,250		618,290		592,007		572,010		517,180		497,901		
Pregnancy loss	78,955	(13.7)	78,178	(13.8)	88,119	(14.3)	86,359	(14.6)	87,418	(15.3)	85,051	(16.5)	87,047	(17.5)	
Age (years)															<.0001
20–24	48,486	(8.4)	42,626	(7.5)	45,055	(7.3)	41,132	(7.0)	38,409	(6.7)	34,585	(6.7)	32,426	(6.5)	
25–29	245,572	(42.7)	240,443	(42.3)	240,601	(38.9)	218,149	(36.9	189,836	(33.2)	158,018	(30.6)	141,541	(28.4)	
30–34	212,035	(36.9)	210,646	(37.1)	243,981	(39.5)	243,793	(41.2)	252,410	(44.1)	236,803	(45.8)	235,983	(47.4)	
35–39	59,794	(10.4)	64,083	(11.3)	75,969	(12.3)	76,053	(12.9)	76,989	(13.5)	74,209	(14.4)	73,782	(14.8)	
40–44	8914	(1.6)	9826	(1.7)	12,037	(2.0)	12,154	(2.1)	13,666	(2.4)	12,850	(2.5)	13,442	(2.7)	
45–49	581	(0.1)	626	(0.1)	647	(0.1)	726	(0.1)	700	(0.1)	715	(0.1)	727	(0.2)	
Income^b^															<.0001
Q0 (lowest)	4202	(0.7)	4378	(0.8)	5028	(0.8)	4654	(0.8)	4271	(0.8)	3456	(0.7)	3063	(0.6)	
Q1	110,516	(19.2)	108,177	(19.0)	116,866	(18.9)	106,348	(18.0)	99,512	(17.4)	88,434	(17.1)	82,476	(16.6)	
Q2	163,980	(28.5)	160,661	(28.3)	179,220	(29.0)	167,838	(28.4)	156,689	(27.4)	140,627	(27.2)	135,528	(27.2)	
Q3	193,465	(33.6)	193,068	(34.0)	209,500	(33.9)	204,715	(34.6)	202,021	(35.3)	184,480	(35.7)	178,350	(35.8)	
Q4 (highest)	103,219	(17.9)	101,966	(17.9)	107,676	(17.4)	108,452	(18.3)	109,517	(19.2)	100,183	(19.4)	98,484	(19.8)	

N: Number

*P* value was calculated with Chi-square test ^a^ P for trend was calculated with Cochran-Armitage test

^b^ Q0: Medical aid beneficiaries, Q1: The lower class (75–100%), Q2: The lower-middle classes (50–74%), Q3: The upper-middle classes (25–49%), Q4: The upper class (1–24%)

**Fig. 1:**
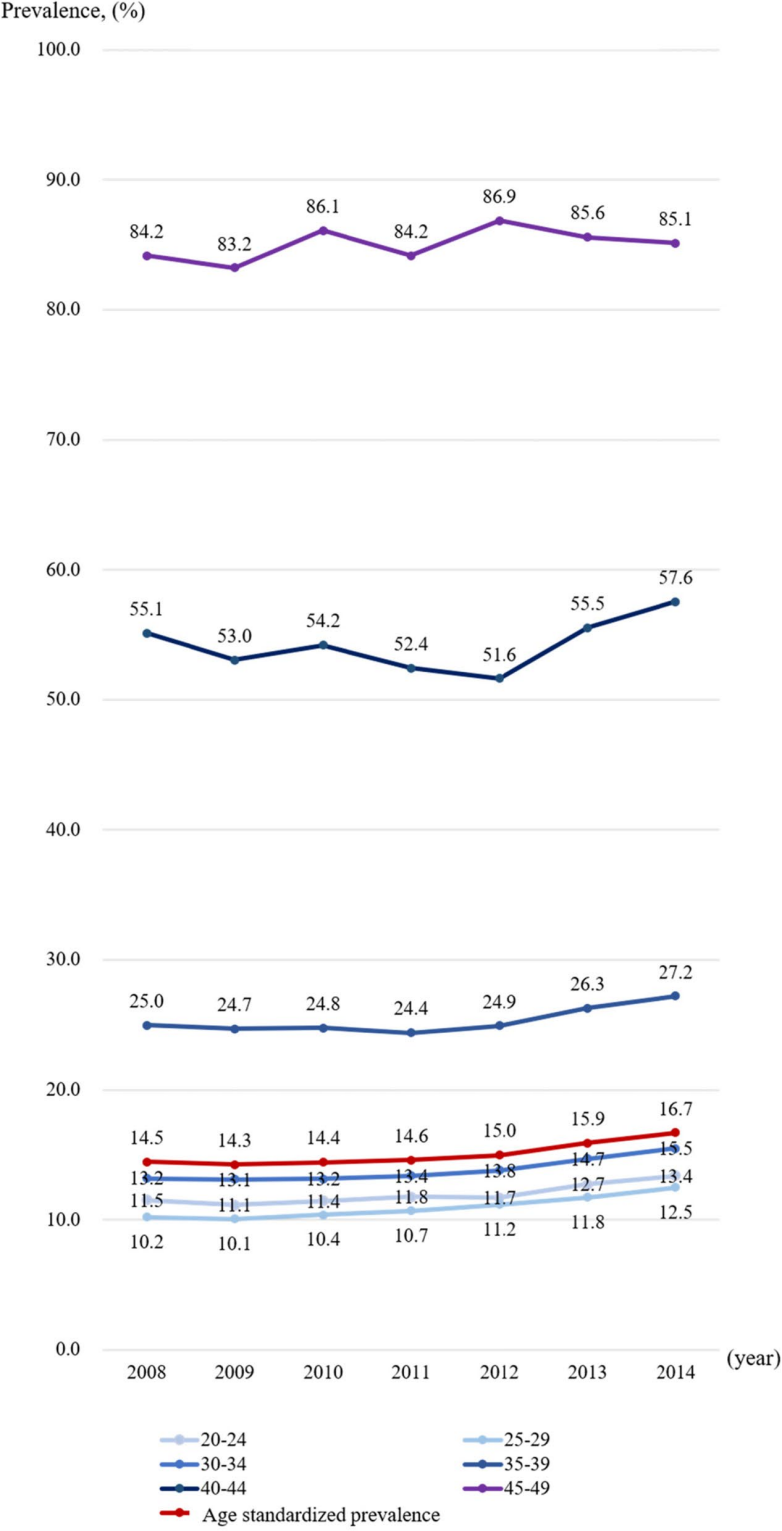
Age-specific annual prevalence and age-standardized annual prevalence of pregnancy loss between 2008 and 2014

Age-specific annual prevalence of pregnancy loss and direct age-standardized annual prevalence of pregnancy loss from 2008 to 2014 are plotted in Fig. 1.

Table 2 shows the numbers, average yearly prevalence, and pregnancy loss ratios according to age and income levels for seven years (from 2008 to 2014). The average annual prevalence among 35–39 years old was 25.3%, which was around twice as high compared to those under the age of 34 years. The average annual prevalence of pregnancy loss increased sharply with age (54.2%, 40–44 years; 85.1%, 45–49 years). The prevalence ratios were also significantly higher in all other age groups compared to the reference (25-29 years) age group, and a positive association was found to increase with age over 25 years.

**Table 2:** The average annual prevalence of pregnancy loss and prevalence ratio according to age and income levels from 2008 to 2014

	N	P	PR	95% CI		adj.PR^a^	95% CI	
Age (years)								
20 ~ 24	33,570	11.9	1.09	1.08	1.11	1.08	1.06	1.10
25 ~ 29	155,029	10.8	Ref.			Ref.		
30 ~ 34	226,612	13.9	1.28	1.27	1.29	1.28	1.27	1.29
35 ~ 39	126,951	25.3	2.34	2.32	2.36	2.31	2.28	2.33
40 ~ 44	44,948	54.2	5.01	4.94	5.08	4.84	4.77	4.91
45 ~ 49	4017	85.1	7.86	7.55	8.20	7.54	7.24	7.86
Income^b^								
Q0 (lowest)	6608	22.7	1.59	1.54	1.64	1.41	1.36	1.45
Q1	109,689	15.4	1.07	1.06	1.08	1.11	1.10	1.12
Q2	156,313	14.2	0.99	0.98	1.00	1.05	1.04	1.06
Q3	195,059	14.3	Ref.			Ref.		
Q4 (highest)	123,458	16.9	1.18	1.17	1.19	1.06	1.05	1.07

*N*: Number, P: Annual specific prevalence for 7 years from 2008 to 2014 (%), *PR* Prevalence ratio, *CI* Confidence interval, *Ref* Reference

^a^ Adjusted for income levels and type of medical insurance qualification for comparing age groups, and adjusted for age and type of medical insurance qualification for comparing income levels

^b^ Q0: Medical aid beneficiaries, Q1: The lower class (75–100%), Q2: The lower-middle classes (50–74%), Q3: The upper-middle classes (25–49%), Q4: The upper class (1–24%)

For income levels, the average annual prevalence and prevalence ratio of pregnancy loss were highest in the medical aid benefit recipient group (Q0), which had the highest increase in risk of prevalence ratio (41%) compared to the Q3 income group (reference). As the income level increased up to Q3, the risk of pregnancy ratio decreased, and the risk increased slightly in the Q4 group. Similar results were found with adjustment for age and type of medical insurance qualification.

The trend was examined using the joint-point analysis to identify changes in age-standardized annual prevalence of pregnancy loss from 2008 to 2014 (Table 3). The crude annual percent change (APC) of age-standardized annual prevalence of pregnancy loss showed no significant change from 2008 to 2011, while it showed a significant annual increase of 4.8% from 2011 to 2014. After adjustment for income levels, the APC showed a significant increase of 5.8% per year from 2011 to 2014. Even if adjusted age and income, the increase in pregnancy loss stood out during 2011–2014 period.

**Table 3:** Trends for the annual prevalence of pregnancy loss with join-point analysis between 2008 and 2014

	2008 - 2011		2011 - 2014		Overall
	Period	APC^b^	Period	APC	AAPC^c^
ASP^a^	2008-2011	0.2	2011-2014	4.8^d^	2.5^d^
Adjusted for income levels	2008-2011	-0.4	2011-2014	5.8^d^	2.6^d^

^a^*ASP* Direct age-standardized annual prevalence between 2008 and 2014

^b^*APC* Annual percent change of age standardized prevalence

^c^*AAPC* average annual percent change

^d^indicates that the annual percent change is significantly different from zero at the alpha = 0.05 level

## Discussion

This study examined the national prevalence and trends of pregnancy loss among Koreans aged 20–49 years from 2008 to 2014 in the Republic of Korea. We also identified the risk of pregnancy loss across income levels as prevalence ratios.

The average annual prevalence for seven years (from 2008 to 2014) was 15.0%. This is similar to the results of other countries: 13.5% in Denmark [7], 18.2% in the United States [9], 10.6% in Jerusalem [31], and 12.2% in Italy [8].

Consistent with other studies, it was found that the risk of pregnancy loss increased with gestational age, especially among those aged over 35 years. The prevalence ratio increased more than twice after the age of 35–39 years, and the prevalence ratio reached up to 7.9 at the age of 45–49 years. We included only those cases wherein pregnancy diagnosis and treatment were confirmed based on medical service use data. Thus, there is a possibility that greater pregnancy loss was reported for the older population because assisted reproductive technology (used more by the older adults) could be reported more accurately by the medical insurance code. Through the analysis of age distribution for some of the excluded population, it was found that the exclusion rate over aged 35 increased as the age increased (data not shown). Considering the high pregnancy loss rate over 35 year of age, it could be suggested that the estimated pregnancy rate has been underestimated in spite of the possible selection bias.

The annual pregnancy loss significantly increased from 13.7% in 2008 to 17.5% in 2014. The APC of age-standardized prevalence (ASP) of pregnancy loss (between 2008 and 2014), increased significantly by 2.5% every year since 2011. When income level was adjusted, ASP significantly increased by 2.6% every year since 2011. These trends could be observed at all age specific annual pregnancy loss prevalences. In particular, the increasing APC during the 2011–2014 period was significantly prominent even after considering the age and income level. The influence of ecologic factors such as air pollution might be suggested [32], but attention should be paid to interpretation such as short observation periods and the selection bias.

Low income has been an influencing factor in other negative pregnancy outcomes such as premature birth, stillbirth, and infant mortality in addition to spontaneous abortion [23, 33–35]. Our study also showed that the risk of pregnancy loss according to income level was higher in all income groups compared to the Q3 income group. The income level and risk of pregnancy loss were inversely proportional among the population under the Q3 income group. Moreover, the risk increased to 41% in the Q0 (lowest income) group adjusted for age and type of medical insurance qualification. Considering Western research, a study on the relationship between socioeconomic statuses of women and spontaneous abortion incidence based on the Danish national birth cohort showed that the lower the income quintiles, the higher was the spontaneous abortion incidence [23]. A large population-based cross-sectional study in China also found a lower prevalence of spontaneous abortion with higher income (compared to the lower-income groups) [36]. There have been studies in Korea confirming differences in infant mortality and childbirth outcomes according to parents’ educational and occupational levels [37]. However, as far as we know, this is the first study to identify the income inequality and trends of pregnancy loss in the Republic of Korea. Recently, one study has been published on socioeconomic status and pregnancy outcomes using the national health insurance data, but only 2010 data were used [38]. Herein, the slightly higher risk of pregnancy loss in the highest income group might be because assisted reproductive technology, which impose a risk for pregnancy loss, was available more easily to the highest income population. Psychosocial stresses due to socioeconomic inequality, low body mass index, inadequate weight gain, and malnutrition are associated with increased negative pregnancy outcomes; therefore, these risk factors are considered the outcomes of low socioeconomic levels. It is necessary to further study whether socioeconomic status is mediated by other factors or independently influences pregnancy loss.

While the interpreting the results of this study, it should be noted that clinically unidentifiable cases might be unreported due to early miscarriage and lack of self-recognition for pregnancy, which is a limitation of medical insurance claim data. Additionally, the number of pregnancy losses among operationally defined pregnancy cases was calculated without considering the course of pregnancy; thus, clearly distinguishing spontaneous abortion and stillbirth was not possible. It was presumed that most pregnancy losses were miscarriages because stillbirth (death of fetus over 22–24 gestational weeks) had low prevalence in high-income countries [39]. However, the longitudinal studies from the beginning till the end of pregnancy are needed. Unlike previous studies, we did not consider important risk factors of miscarriage. It was not known whether the subject had miscarriage; moreover, the pregnancy history was also not known. If systematic misclassification bias of recurrent miscarriages is not be assumed, the results have not been overestimated. Nevertheless, the findings of this study may have appreciable significance because national representative population-based data were analyzed. Further, pregnancy loss variables were detected more precisely using the NHIS as compared to previous self-reported surveys.

## Conclusion

This would be the foundational epidemiologic information on the current status and trend of pregnancy loss in the Republic of Korea. Inequalities in pregnancy loss across income levels could also be identified. Income inequality in pregnancy loss is also observed even in high-income countries with national health insurance service. Further appraisal is needed to explain the increasing trend of pregnancy loss between 2011 and 2014 even after adjusting income. There is a need for a comprehensive policy that includes a detailed monitoring and evaluation for protection of women’s reproductive and sexual health.

## Data Availability

The data that support the findings of this study are available from Korea National Health Insurance but restrictions apply to the availability of these data, which were used under license for the current study, and so are not publicly available. Data are however available from the authors upon reasonable request and with permission of Korea National Health Insurance.
